# Metabolic response prediction using ^68^Ga-FAPI PET/CT in Non-Hodgkin lymphoma treated with chemotherapy: a pilot study

**DOI:** 10.1186/s40644-025-00890-0

**Published:** 2025-06-08

**Authors:** Linwei Li, Hongyin Ding, Lingzhi Chen, Dengsai Peng, Yue Chen

**Affiliations:** 1 Department of Nuclear Medicine, The Affiliated Hospital of Southwest Medical University, Luzhou, Sichuan 646000 China; 2 Nuclear Medicine and Molecular Imaging Key Laboratory of Sichuan Province, No 25 TaiPing St, Jiangyang District, Luzhou, Sichuan 646000 China; 3 Institute of Nuclear Medicine, Southwest Medical University, Luzhou, Sichuan 646000 China; 4 Department of Ultrasound, The Affiliated Hospital of Southwest Medical University, Luzhou, Sichuan 646000 China

## Abstract

**Background:**

The aim of this study was to investigate the prediction value of metabolic response using gallium 68 (^68^Ga) labeled fibroblast-activation protein inhibitor (^68^Ga-FAPI) positron emission tomography-computed tomography (PET/CT) in Non-Hodgkin lymphoma (NHL) patients receiving (cyclophosphamide-doxorubicin HCl-vincristine[Oncovin]- prednisone) CHOP-like chemotherapy.

**Method:**

This single-center prospective study was conducted in our hospital and enrolled participants who was initially diagnosed with NHL and received CHOP-like chemotherapy. ^68^Ga-FAPI PET/CT was performed before chemotherapy. Metabolic response was assessed by fluorine 18 (^18^F) labeled fluorodeoxyglucose (^18^F-FDG) PET/CT. Quantitative analysis included measurement of the maximum standardized uptake value (SUVmax), mean standardized uptake value (SUVmean), peak standardized uptake value (SUVpeak), metabolic tumor volume (MTV) and total lesion FAP (TLF). The SUVmax value of the lesion is divided by SUVmean of normal tissue to calculate the target-to-background ratio (TBRblood and TBRmuscle). Depending on the response, participants were categorized as responders and non-responders. Mann-Whitney U-test was used to compare the ^68^Ga-FAPI PET/CT parameters of responders with that of non-responders. Logistic regression analyses were performed to determine the relationship between clinical characteristics, ^68^Ga-FAPI PET/CT parameters, and efficacy of chemotherapy. Receiver operating characteristic curve analysis was used to identify the accuracy of ^68^Ga-FAPI PET/CT parameters for response prediction.

**Results:**

From October 2022 to May 2023, 18 participants (10 men and 8 women; median age: 56 years [interquartile range: 47–67 years]) with pathologically confirmed diagnosis of non-Hodgkin’s lymphoma were recruited in our hospital and enrolled in this study. The mean values of SUVmax, TBRblood, and TBRmuscle were significantly higher in responders than those in non-responders (8.41$\:\pm\:$3.90 vs. 3.98$\:\pm\:$2.81 *P=*0.025; 7.93$\:\pm\:$3.31 vs. 3.69$\:\pm\:$2.36 *P=*0.035; 7.04$\:\pm\:$3.22 vs. 3.09$\:\pm\:$1.73 *P =* 0.025; respectively). The area under the curve (AUC) of SUVmax, TBRblood, and TBRmuscle were statistically significant (0.875, *P =* 0.025; 0.857, *P=*0.034; 0.875, *P =* 0.026, respectively). SUVmax (OR=0.592, *P* = 0.041) is a significant factor in the prognosis of these participants.

**Conclusion:**

Low radiotracer uptake on ^68^Ga-FAPI PET/CT indicated poor metabolic response of NHL patients received CHOP-like therapy. SUVmax could be used to screen sensitive patients.

## Introduction

Non-Hodgkin lymphoma (NHL) is a heterogeneous group of diseases that constitute the most diagnosed hematological malignancies worldwide [1]. NHL is the 10th most common and 11th deadliest malignancy, according to 2022 GLOBAN [2]. By far the most common NHL subtypes are diffuse large B-cell lymphoma and follicular lymphoma. The advent of rituximab was one of the most important milestones in the treatment of NHL, and R-CHOP (rituximab-cyclophosphamide-doxorubicin HCl-vincristine[Oncovin]- prednisone) is the first-line chemotherapy regimen for mostly NHL [3–5]. However, part of patients (47% in peripheral t-cell lymphoma, for example) is refractory to first-line chemotherapy and resistance is associated with poor outcomes with short survival [6].

Fibroblast activation protein (FAP) is a type II transmembrane serine protease. Gallium 68 (^68^Ga) labeled fibroblast-activation protein inhibitor (^68^Ga-FAPI) positron emission tomography-computed tomography (PET/CT) has been used to image a wide range of tumors and promises to be a broad-spectrum imaging agent [7, 8]. A recent study has shown that ^68^Ga-FAPI imaging can be used to detect FAP expression in lymphoma lesions [9]. However, several previous studies have demonstrated that ^68^Ga-FAPI PET/CT has poorer diagnostic efficacy than fluorine 18 (^18^F) labeled fluorodeoxyglucose (^18^F-FDG) PET/CT for lymphoma, especially those with low FAP expression [10–12]. In addition, the knowledge of ^18^F-FDG utility in evaluating the efficacy after treatment is well-established. Comparing metabolic activity changes of tumor to evaluate the efficacy of treatment is an important clinical approach [13]. Although ^18^F-FDG remains the gold standard, ^68^Ga-FAPI can noninvasively reveal the tumor microenvironment, which may have implications for tumor prognosis. The FAP-mediated tumor microenvironment plays a key role in drug resistance of tumor cells [14, 15]. Several studies have demonstrated that ^68^Ga-FAPI parameters at baseline can be used for the prediction of treatment response in tumors, but they are mainly limited to digestive tumors [16–19]. By far, whether FAPI can be used for the prediction of efficacy of chemotherapy in lymphomas remains unclear.

The aim of this study was to investigate the prediction value of metabolic response using FAPI PET/CT in lymphoma patients receiving CHOP-like chemotherapy.

## Materials and methods

### Participants

This single-center prospective study was conducted in our hospital and enrolled participants who was initially diagnosed with NHL from Oct. 2022 to May. 2023. The clinical trial was approved by the hospital’s ethics committee and followed the 1964 Helsinki Declaration. All recruited participants volunteered for this study and signed an informed consent form. Inclusion criteria included: pathologically confirmed non-Hodgkin’s lymphoma; presence of at least one measurable solid target tumor; willingness to undergo chemotherapy and ^68^Ga-FAPI PET/CT. Exclusion criteria included unwillingness to undergo examination or treatment; younger than 18 years of age; pregnancy or lactating.

### Chemotherapy and metabolic response assessment

All participants started chemotherapy within 1 week after the baseline ^18^F-FDG PET/CT. Deauville score was attained by an experienced nuclear medicine physician. Each participant received monthly chemotherapy for a total of 4 cycles, and ^18^F-FDG PET/CT was performed again within 1 week after the end of treatment. Response was assessed with reference to the Positron Emission Tomography (PET) Response Criteria in Solid Tumors (PERCIST 1.0), which defined response as complete metabolic response (CMR), partial metabolic response (PMR), stable metabolic disease (SMD) and progressive metabolic disease (PMD) [20]. Briefly, CMR = metabolic activity of target lesion decreased below the liver background, PMR = peak standardized uptake lean (SULpeak) of target lesion decreases to less than 30% or, alternatively, absolute SULpeak decreases greater than 0.8 without newly lesions appear, PMD = SULpeak of target lesion increases to more than 30% or, alternatively, absolute SULpeak increases greater than 0.8 or there are newly lesions. SMD = not met with CMR, PMR, or PMD. Depending on the response, participants were categorized as responders (CMR or PMR) and non-responders (SMD or PMD).

### ^68^Ga-FAPI PET/CT acquisition

FAPI-04 was purchased from MCE (MedChemExpress, USA) with a purity of 98%. 4 mL of ^68^Ga solution (1.7 GBq) and 50 µg of FAPI-04 were dissolved in 1 mL of sodium acetate solution (0.25 M) with a pH of 3.3–3.6. The reaction was heated at 80 °C for 10 min. The reaction was heated at 80 °C for 10 min and the product was purified using a Seppak 18 C chromatographic column, followed by elution with 1 mL of 50% ethanol and 4 mL of physiological saline. Quality control was assessed by radio-high performance liquid chromatography (HPLC) with radiochemical purity exceeding 98%.

^68^Ga-FAPI PET/CT was performed using a United imaging system. Participants received ^68^Ga-FAPI-04 (0.05 mCi/kg) through an intravenous indwelling line and were imaged 50–60 min after injection. Whole-body 3D PET scans were performed from the base of the skull to the mid-thighs using 5 to 6 beds (3 min/bed), with a layer thickness of 3 mm and a matrix of 128 × 128. All PET images were attenuated and iteratively reconstructed from CT scans.

### Imaging interpretation

^68^Ga-FAPI PET/CT images were visually assessed by two experienced nuclear medicine physicians (two-years experience in ^68^Ga-FAPI imaging). All disagreements were solved in consensus after discussion. Quantitative analysis included measurement of the maximum standardized uptake value (SUVmax), mean standardized uptake value (SUVmean), peak standardized uptake value (SUVpeak), metabolic tumor volume (MTV) and total lesion FAP (TLF). Each value was calculated as the mean of three measurements using a spherical volume of interest (VOI) of 3 cm in diameter placed at the lesion. Background activity was measured using SUVmean in ROIs of 1 cm diameter at the pulmonary artery trunk and erector spinae. The SUVmax value of the lesion is divided by these SUVmean value to calculate the target-to-background ratio (TBRblood and TBRmuscle). If there were multiple lesions at one site, the average value was used, which was defined as averaging the parameters of all lesions (no more than 5 lesions) or averaging the SUVmax of 5 biggest lesions ranked by their longer length (more than 5 lesions).

### Statistical analysis

Statistical analyses were performed using SPSS statistical software (version 26.0; IBM). Quantitative data including SUVmax, SUVmean, SUVpeak, MTV, TLF, TBRblood, and TBRmuscle are expressed as mean $\:\pm\:$ SD. Mann-Whitney U-test was used to compare the ^68^Ga-FAPI PET/CT parameters of responders with that of non-responders. Logistic regression analyses were performed to determine the relationship between baseline Deauville score, initial staging, ^68^Ga-FAPI PET/CT parameters, and efficacy of chemotherapy. Receiver operating characteristic curve analysis was used to determine the cut-off value based on maximum Youden index and the accuracy of ^68^Ga-FAPI PET/CT parameters for response prediction. The threshold of statistical significance P value was set at 0.05.

## Results

### Participants cohort

**Fig. 1:**
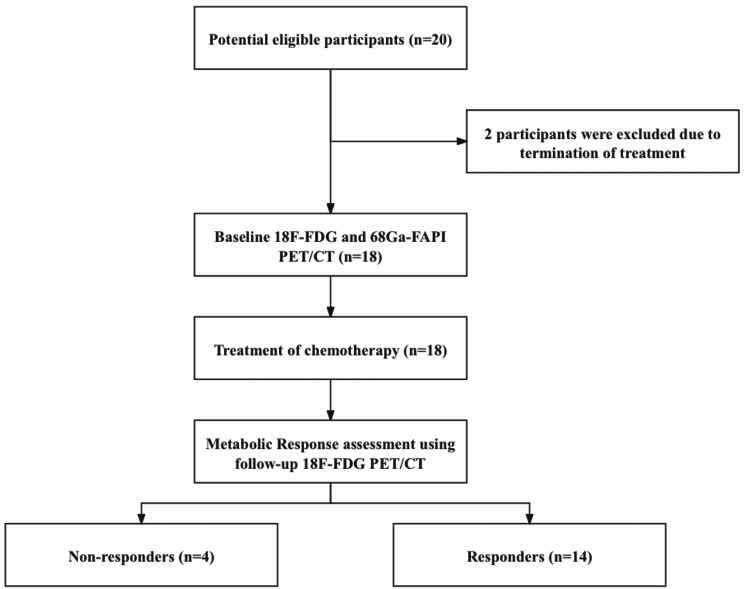
Flowchart of this study. ^18^F-FDG = fluorine 18 (^18^F) labeled fluorodeoxyglucose; ^68^Ga-FAPI = gallium 68 (^68^Ga) labeled fibroblast-activation protein inhibitor; PET/CT = positron emission tomography-computed tomography

From October 2022 to May 2023, 18 participants (10 men and 8 women; median age: 56 years [interquartile range: 47–67 years]) with pathologically confirmed diagnosis of non-Hodgkin’s lymphoma were recruited in our hospital and enrolled in this study. The flow chart is shown in Fig. 1. Characteristics of each participant were shown in Table 1. In summary, 14 participants were responders, and 4 participants were non-responders.

**Table 1:** Characteristics of participants

No.	Sex	Age	Type	Stage	KPS	Deauville score (Baseline)	Chemotherapy	Response
1	M	49	AITL	IIIA	90	5	CHOP	PMR
2	M	54	DLBCL	IIB	90	5	CHOP	CMR
3	M	69	MCL	IVA	90	5	CHOP	PMD
4	M	68	DLBCL	IIA	90	5	R-CHOP	PMR
5	M	22	DLBCL	IVA	90	5	CHOP	CMR
6	M	54	DLBCL	IVA	90	5	R-CHOP	PMR
7	F	67	DLBCL	IIIA	80	5	R-CHOP	PMD
8	F	60	DLBCL	IIA	90	5	R-CHOP	CMR
9	F	81	ALCL	IIIA	90	5	CHOP	PMR
10	M	69	DLBCL	IIIS	80	5	CHOP	CMR
11	F	57	MCL	IVB	90	5	R-CHOP	PMR
12	M	58	MCL	IVB	90	3	R-CHOP	SMD
13	F	53	MCL	IVA	90	5	R-CHOP	SMD
14	M	47	DLBCL	IIIA	90	5	R-CHOP	CMR
15	F	65	DLBCL	IIIA	90	5	R-CHOP	PMR
16	F	46	DLBCL	IVA	90	5	CHOP	CMR
17	M	32	PTCL	IIIA	90	5	CHOP	CMR
18	F	22	FL	IIA	90	5	R-COP	CMR

AITL: angioimmunoblastic T-cell lymphoma; ALCL: anaplastic large cell lymphoma; MCL: mantle cell Lymphoma; DLBCL: diffuse large B-cell lymphoma; FL: follicular lymphoma; PTCL: peripheral T-cell lymphoma; CMR = complete metabolic response; PMR = partial metabolic response; SMD = stable metabolic disease; PMD = progressive metabolic disease; R-CHOP (rituximab-cyclophosphamide-doxorubicin HCl-vincristine[Oncovin]- prednisone); KPS: Karnofsky Performance Status

### ^68^Ga-FAPI PET/CT parameters comparison

**Fig. 2:**
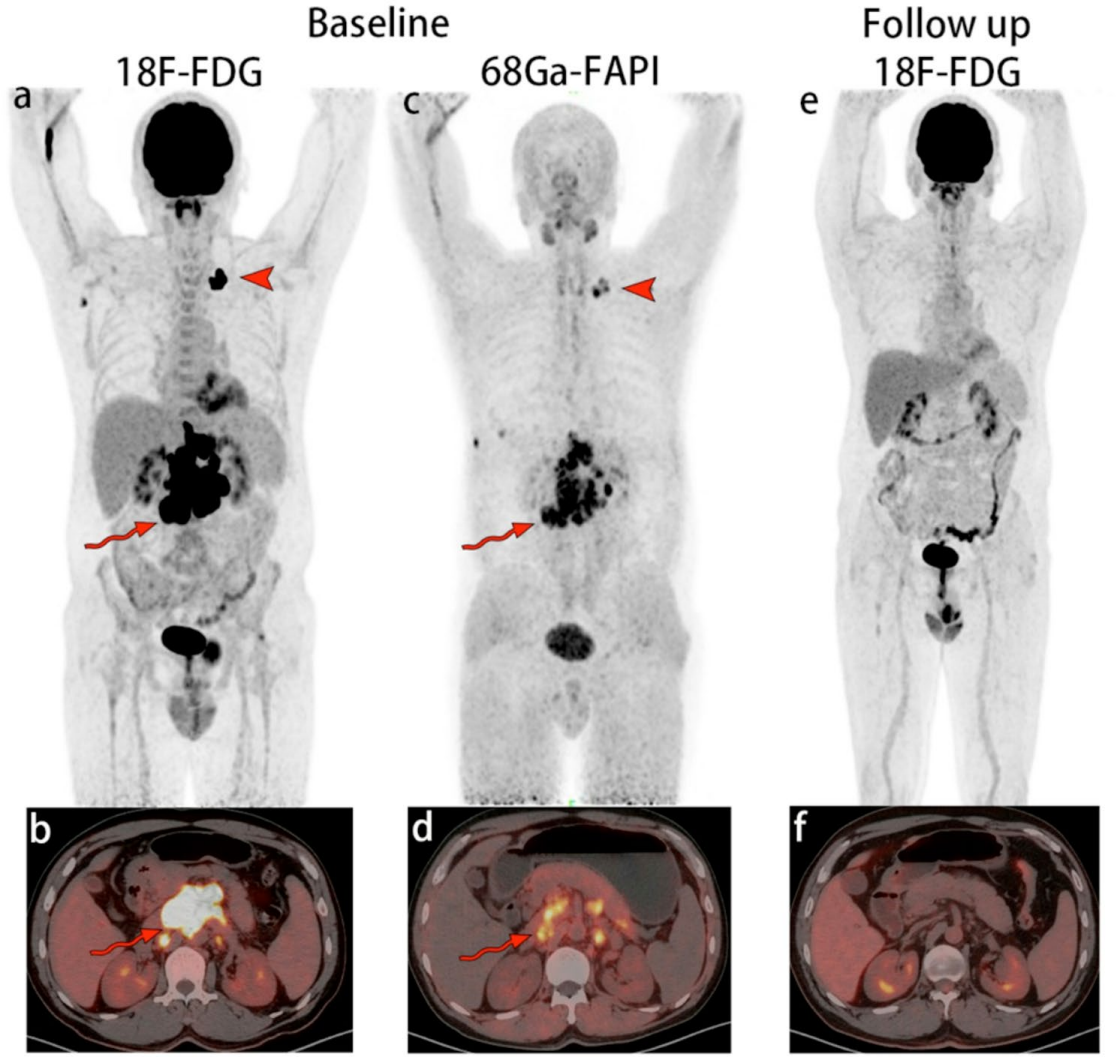
Images of a participant with aggressive large B-cell lymphoma confirmed by biopsy. Received 4 cycles of CHOP chemotherapy. The maximum intensity projection (MIP) image and fusion image of baseline ^18^F-FDG PET/CT showed increased radiotracer uptake of lymph nodes in the abdomen and mediastinum (**a**, **b**, curved arrows/arrowhead, SUVmax = 21.0). These lesions were also detected on ^68^Ga-FAPI PET/CT (**c**, **d**, curved arrows/arrowhead, SUVmax = 8.0). After 4 cycles of CHOP chemotherapy. A CMR metabolic response were assessed by follow-up ^18^F-FDG PET/CT (**e**, **f**. ^18^F-FDG = fluorine 18 (^18^F) labeled fluorodeoxyglucose; ^68^Ga-FAPI = gallium 68 (^68^Ga) labeled fibroblast-activation protein inhibitor; PET/CT = positron emission tomography-computed tomography

The ^68^Ga-FAPI-04 PET/CT quantitative baseline parameters SUVmax, SUVmean, SUVpeak, MTV, TLF, TBRblood, and TBRmuscle for all patients, responders, and non-responders are listed in Table 2. Images of a representative participants were shown in Fig. 2. The mean values of SUVmax, TBRblood, and TBRmuscle were significantly higher in responders than those in non-responders (8.41$\:\pm\:$3.90 vs. 3.98$\:\pm\:$2.81 *P=*0.025; 7.93$\:\pm\:$3.31 vs. 3.69$\:\pm\:$2.36 *P=*0.035; 7.04$\:\pm\:$3.22 vs. 3.09$\:\pm\:$1.73 *P =* 0.025; respectively) (Table 2). None of the other parameters were significantly different between responders and non-responders.

**Table 2:** ^68^Ga-FAPI PET/CT parameters comparison

Parameters	All participants (*n* = 18)	responders (*n* = 14)	non-responders(*n* = 4)	*P*
SUVmax	7.42$\:\pm\:$4.08	8.41$\:\pm\:$3.90	3.98$\:\pm\:$2.81	0.025
SUVmean	4.05$\:\pm\:$2.05	4.41$\:\pm\:$2.01	2.76$\:\pm\:$1.86	0.158
TLF	85.40$\:\pm\:$112.86	97.68$\:\pm\:$125.01	42.41$\:\pm\:$35.89	0.233
SUVpeak	5.40$\:\pm\:$2.64	5.84$\:\pm\:$2.60	3.85$\:\pm\:$2.44	0.192
MTV	20.38$\:\pm\:$20.98	21.16$\:\pm\:$22.53	20.15$\:\pm\:$17.10	0.721
TBRblood	6.99$\:\pm\:$3.56	7.93$\:\pm\:$3.31	3.69$\:\pm\:$2.36	0.035
TBRmuscle	6.17$\:\pm\:$3.36	7.04$\:\pm\:$3.22	3.09$\:\pm\:$1.73	0.025

SUV_max_ = maximum standardized uptake value; SUV_mean_ = mean standardized uptake value; SUV_peak_ = peak standardized uptake value; MTV = metabolic tumor volume; TLF = total lesion fibroblast-activation protein; TBR = tumor-to-background ratio

### Receiver operating characteristic (ROC) curves analysis

**Fig. 3:**
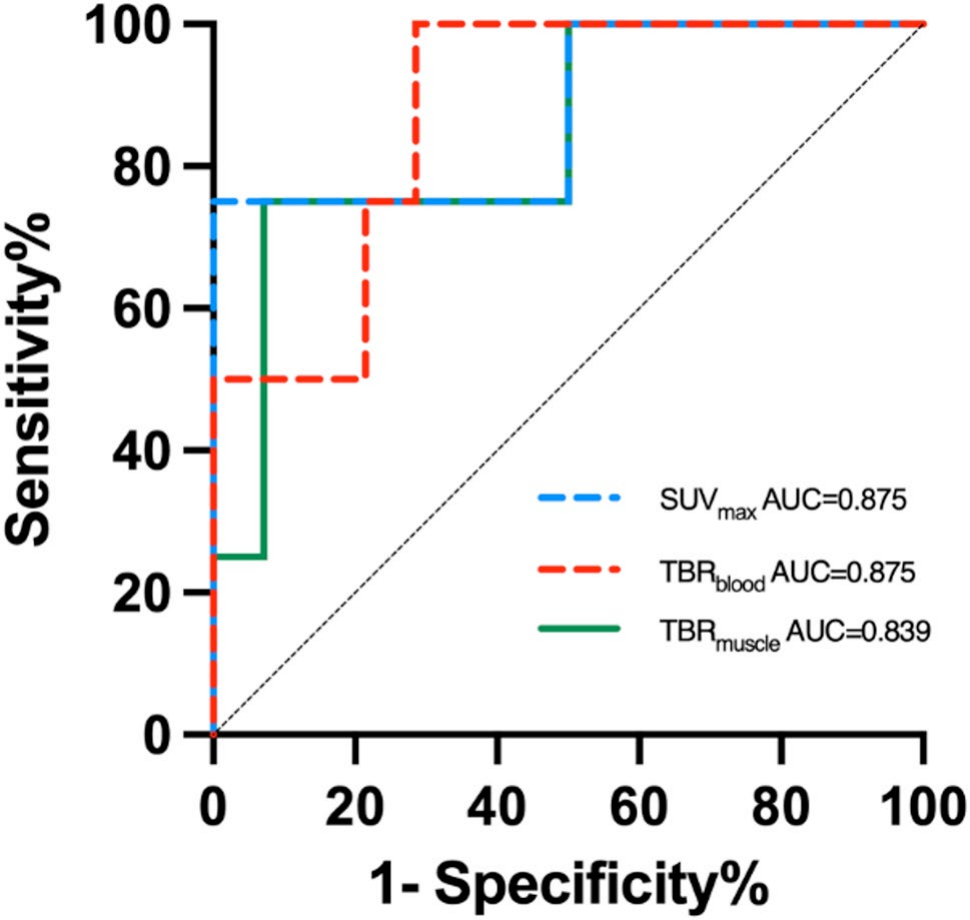
Receiver-operating-characteristic curves of parameters to predict metabolic response. SUV_max_ = maximum standardized uptake value; TBR = tumor-to-background ratio

ROC curves were created to determine the accuracy of ^68^Ga-FAPI PET/CT parameters in identifying responders and non-responders. The area under the curve (AUC) of SUVmax, TBRblood, and TBRmuscle were statistically significant (0.875, *P =* 0.025; 0.857, *P =* 0.034; 0.875, *P =* 0.026, respectively) (Table 3; Fig. 3). The cutoff value (based on Youden index) of SUVmax, TBRblood, and TBRmuscle were 3.6, 4.4, and 4.1. The sensitivity and specificity of SUVmax, TBRblood, and TBRmuscle were 92.9% and 75% when using respective cutoff value.

**Table 3:** AUC of ^68^Ga-FAPI PET/CT parameters

Parameters	Area	Std. Error a	Asymptotic Sig. b	Asymptotic 95% Confidence Interval	
				Lower Bound	Upper Bound
SUVmax	0.875	0.117	0.026	0.646	1
SUVmean	0.75	0.154	0.137	0.448	1
TLF	0.714	0.157	0.203	0.406	1
SUVpeak	0.732	0.153	0.167	0.432	1
MTV	0.429	0.15	0.671	0.134	0.723
TBRblood	0.857	0.131	0.034	0.6	1
TBRmuscle	0.875	0.093	0.026	0.693	1
Under the nonparametric assumption					
Null hypothesis: true area = 0.5					

SUV_max_ = maximum standardized uptake value; SUV_mean_ = mean standardized uptake value; SUV_peak_ = peak standardized uptake value; MTV = metabolic tumor volume; TLF = total lesion fibroblast-activation protein; TBR = tumor-to-background ratio

### Correlation between parameters

**Fig. 4:**
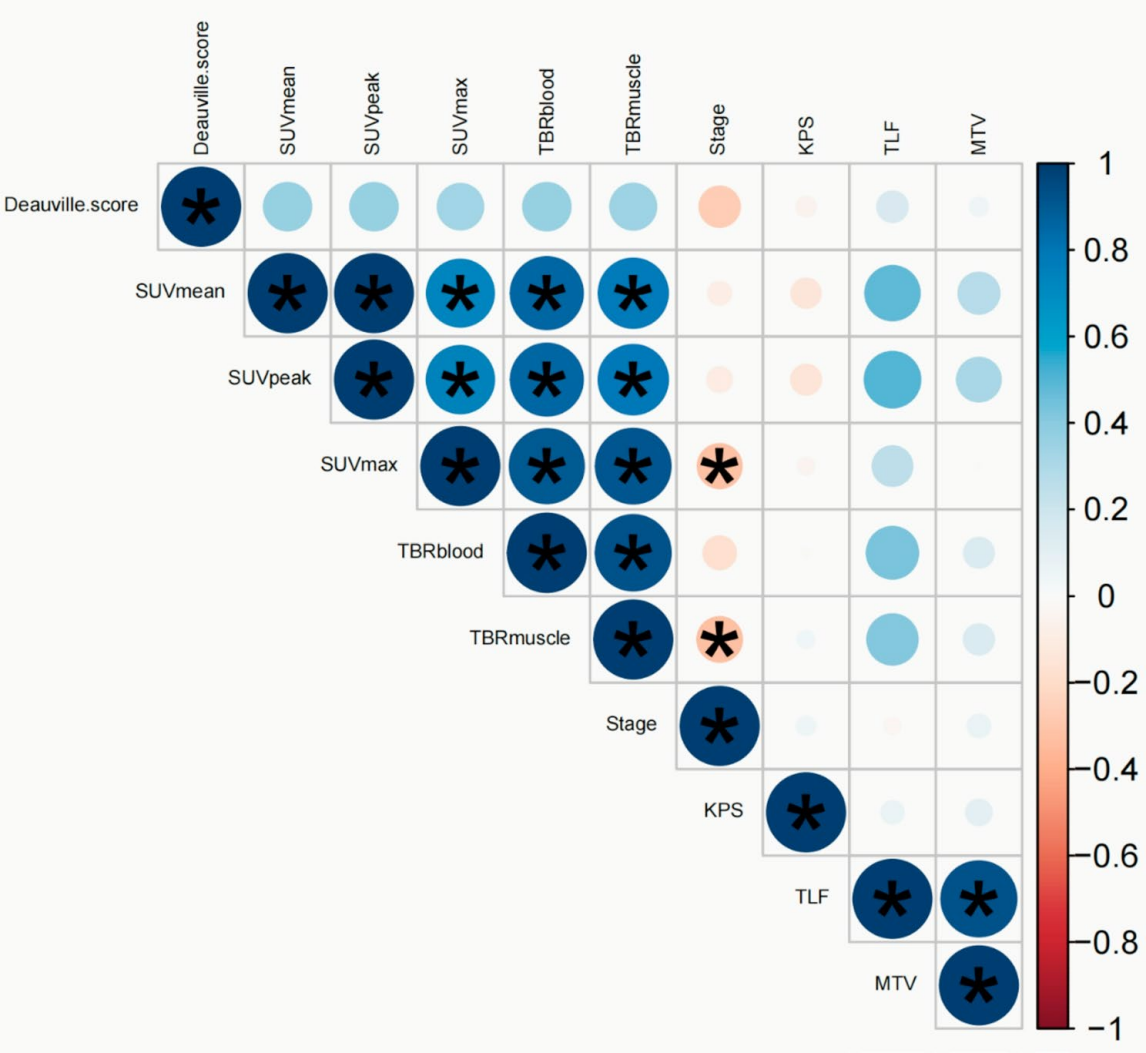
Correlations between ^68^Ga-FAPI PET/CT parameters and clinical variables. Those with statical significance are marked by asterisks. Blue represents positive correlation between 2 variables, and red represents negative correlation. The stronger the correlation, the darker the color. SUV_max_ = maximum standardized uptake value; SUV_mean_ = mean standardized uptake value; SUV_peak_ = peak standardized uptake value; MTV = metabolic tumor volume; TLF = total lesion fibroblast-activation protein; TBR = tumor-to-background ratio

SUVmean, SUVpeak, SUVmax, TBRblood, and TBRmuscle correlate with each other significantly, in addition to a weak negative correlation between SUVmax/TBRmuscle and clinical stage (*r*=-0.316, *P* = 0.009; *r*=-0.326, *P* = 0.005). No other parameters have a statistically significant correlation (Fig. 4).

### Association between ^68^Ga-FAPI PET/CT parameters and metabolic response

In univariate logistic regression analyses, SUVmax(OR = 0.780, *P* = 0.019), SUVmean(OR = 0.687, *P* = 0.023), SUVpeak(OR = 0.760, *P* = 0.023), TBRblood(OR = 0.764, *P* = 0.017), and TBRmuscle(OR = 0.735, *P* = 0.02) were associated with metabolic response to chemotherapy in NHL participants. Due to the significant positive correlation between these parameters, we included only SUVmax and stage in the multivariate analysis. Finally, SUVmax (OR = 0.592, *P* = 0.041) remained a significant factor in the prognosis of these participants (Table 4).

**Table 4:** Logistic regression analyses

	Univariate analysis		Multivariate analysis	
Factor	OR(95%CI)	P	OR(95%CI)	P
SUVmax*	0.780(0.635–0.959)	0.019	0.592(0.358–0.978)	0.041
SUVmean*	0.687(0.498–0.949)	0.023	—	—
SUVpeak*	0.760(0.600-0.963)	0.023	—	—
TLF	0.989(0.958–1.020)	0.147	—	—
MTV	0.961(0.910–1.015)	0.154	—	—
TBRblood*	0.764(0.612–0.953)	0.017	—	—
TBRmuscle*	0.735(0.567–0.952)	0.02	—	—
Deauville score	1.326E-10(0.000–1.000)	1	—	—
KPS	0.103(0.000–1.000)	1	—	—
Stage	0.733(0.528–1.017)	0.063	1.705(0.812–3.581)	0.159

SUV_max_ = maximum standardized uptake value; SUV_mean_ = mean standardized uptake value; SUV_peak_ = peak standardized uptake value; MTV = metabolic tumor volume; TLF = total lesion fibroblast-activation protein; TBR = tumor-to-background ratio; KPS: Karnofsky Performance Status

## Discussion

CHOP-like chemotherapy has greatly improved the prognosis of non-Hodgkin’s lymphoma, but there are still some patients who are not sensitive. Therefore, it is even more important to screen patients prior to receiving CHOP-like chemotherapy with validated prognostic markers, thereby accurately guiding on patients’ selection. ^68^Ga-FAPI is a potential radiotracer for tumor stroma visualization that has shown significant value in a wide range of tumors and is a promising alternative to ^18^F-FDG [21–23]. Our study found significantly higher mean values of SUVmax, TBRblood and TBRmuscle in responders compared to non-responders after CHOP-like treatment. The sensitivity and specificity for efficacy prediction could be up to 92.9% and 75%.

Our results showed that NHL patients with higher FAP expression had better CHOP-like chemotherapy efficacy. This may be because lymphomas with higher FAP expression are more aggressive, whereas high-grade lymphomas are usually responded well to chemotherapy [24, 25]. Previously, a correlation between FAPI uptake and clinical classification of NHL has been demonstrated, with aggressive NHL lesions exhibiting moderate to intense FAP immunostaining and ^68^Ga-FAPI uptake. In contrast, indolent NHL lesions exhibit weak FAP staining and mild to moderate ^68^Ga-FAPI uptake [9]. FAP expression in malignant tumors is predominantly expressed by cancer-related fibroblasts (CAFs), which are important regulators of tumor evolution, growth, angiogenesis, immunity, and invasive behaviors (invasion and metastasis) through direct cytokine and extracellular contacts and secretion [26–28].

In addition, better chemotherapeutic outcomes for patients with high FAPI uptake may also be associated with lymphoma stromal fibrosis. Stromal-1 signature have been found that correlate with longer survival in patients with lymphoma treated with R-CHOP [29, 30]. Significant enrichment of “stromal-1” signatures was observed in BCL2(-) diffuse large B-cell lymphoma [31]. This signature includes genes coordinately expressed in many normal mesenchymal tissues, most of which encode proteins that form or modify the extracellular matrix. One of the components of a stroma-1 signature is fibronectin, suggesting that the signature reflects the fibrotic nature of many diffuse large B-cell lymphomas [30]. Whereas ^68^Ga-FAPI targets fibro-activating proteins and therefore may account for the better response to treatment in lymphomas with high FAP uptake.

Some studies have shown the value of baseline ^18^F-FDG to predict prognosis in NHL [32–36]. A meta-analysis have shown that metabolic tumor volume (MTV) is a prognostic predictor, but the threshold for classifying patients into high or low risk groups by MTV is highly dependent on disease classification and treatment [37].

The potential applications of ^68^Ga-FAPI in lymphoma may also include diagnosis and efficacy assessment. Although it has been shown that ^68^Ga-FAPI is poor than ^18^F-FDG for the assessment of most lymphomas, it benefits from the fact that different types or lymphomas with different invasiveness express different proportions of FAP, and the heterogeneity between tumors and foci that it displays may provide help in elucidating interactions in tumor progression [10]. As for some indolent lymphomas such as mucosa-associated lymphoid tissue lymphoma that show poor ^18^F-FDG intensity, ^68^Ga-FAPI will play a greater role in diagnosis, efficacy prediction, and response assessment [38, 39]. Thus, further studies about ^68^Ga-FAPI utility in indolent lymphomas are needed.

Our study has some limitations. First, we included a small case number of patients. Also, we only assessed the relatively short-term response to chemotherapy and did not follow up on survival. Second, the pathological findings of these patients were not consistent. Within each subgroup, there may have been differences in response despite the relative uniformity of treatment regimens. Thirdly, it is possible that FAPI uptake may be different in different subtypes of NHL, but studies with larger samples are still needed.

## Conclusion

This study showed that ^68^Ga-FAPI PET/CT could be used in predicting response of NHL patients to CHOP-like chemotherapy. Low radiotracer uptake on ^68^Ga-FAPI PET/CT indicated poor metabolic response of NHL patients received CHOP-like therapy. SUVmax could be used to screen sensitive patients.

## Data Availability

Data is provided within the manuscript or supplementary information files.

## Abbreviations

NHL: Non-Hodgkin lymphoma
SUV_max_: Maximum standardized uptake value
SUV_mean_: Mean standardized uptake value
SUV_peak_: Peak standardized uptake value
MTV: Metabolic tumor volume
TLF: Total lesion fibroblast-activation protein
TBR: Tumor-to-background ratio
CMR: Complete metabolic response
PMR: Partial metabolic response
SMD: Stable metabolic disease
PMD: Progressive metabolic disease
